# The validation of artificial anti‐monkeypox antibodies by in silico and experimental approaches

**DOI:** 10.1002/iid3.834

**Published:** 2023-04-12

**Authors:** Sadeq Shabani, Mohsen Rashidi, Shakila Radgoudarzi, Ali Jebali

**Affiliations:** ^1^ Department of Biological Sciences Florida International University Miami Florida USA; ^2^ Biomolecular Science Institute Florida International University Miami Florida USA; ^3^ Department Pharmacology, Faculty of Medicine Mazandaran University of Medical Sciences Sari Iran; ^4^ The Health of Plant and Livestock Products Research Center Mazandaran University of Medical Sciences Sari Iran; ^5^ I.M. Sechenov First Moscow State Medical University (Первый МГМУ им) Moscow Russia; ^6^ Department of Medical Nanotechnology, Faculty of Advanced Sciences and Technology, Tehran Medical Science Islamic Azad University Tehran Iran

**Keywords:** anti‐monkeypox antibody, heavy chain, in silico, peptide fragment

## Abstract

As a result of smallpox immunization programs that ended more than 40 years ago, a significant portion of the world's population is not immune. Moreover, due to the lack of anti‐monkeypox drugs and vaccines against monkeypox, the spread of this virus may be the beginning of another challenge. In this study, novel antibodies against monkeypox virus were modeled based on a heavy chain of human antibody and a small peptide fragment. Docking of modeled antibodies with C19L protein showed the range of docking energy, and root‐mean‐square deviation (RMSD) was from −124 to −154 kcal/mL and 4–6 angstrom, respectively. Also, docking of modeled antibodies‐C19L complex with gamma Fc receptor type I illustrated the range of docking energy, and RMSD was from −132 to −155 kcal/ml and 5–7 angstrom, respectively. Moreover, molecular dynamics simulation showed that antibody 62 had the highest stability with the lowest energy level and RMSD. Interestingly, no modeled antibodies had immunogenicity, allergenicity, and toxicity. Although all of them had good stability, only antibodies 25, 28, 54, and 62 had a half‐life of >10 h. Moreover, the interaction between C19L protein and anti‐C19L antibodies (wild‐type and synthetic) was evaluated by the SPR method. We found that K_D_ in synthetic antibodies was lower than wild antibody. In terms of δH°, TδS°, and δG°, the results were consistent with binding parameters. Here, the lowest value of thermodynamic parameters was obtained for antibody 62. These data show that the synthetic antibodies, especially antibody 62, had a higher affinity than the wild‐type antibody.

## Graphical abstract

The three‐dimensional structure model of antibody 25 (A), antibody 28 (B), antibody 32 (C), antibody 37 (D), antibody 54 (E), antibody 62 (F), C19L (G), and gamma Fc receptor type I (H), prepared by SWISS‐model server.

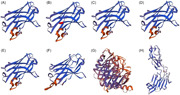

## 1 INTRODUCTION

Recently, the spread of monkeypox in endemic and even non‐endemic areas has attracted international attention.^1^ The monkeypox virus was previously ignored because it was only endemic to West and Central Africa.^2^ The first case in humans was reported in 1970 in the Democratic Republic of Congo.^3^ Over the past 50 years, several thousand human cases have been recorded^4^ in the world. Deforestation, population growth, encroachment on animal habitats, and increased human movement lead to an increase in the virus spread.^5^ This virus belongs to the *Poxviridae* family and has double‐stranded DNA. It can infect a wide range of birds, reptiles, insects, and mammals.^6^, ^7^ *Orthopoxviruses* (mpox) are large viruses with a size of 140–450 nm and a brick‐like structure. They have a genome consisting of 200–500 kbp, which encodes more than 200 genes.^7^ Many of the genes encoded by the mpox genome are not essential for virus replication in cell culture but may play an important role in the host antiviral response.^8^, ^9^ All mpoxs complete their replication cycle in the cytoplasm of infected cells through complex molecular pathways.^10^ The intracellular mature virion and the extracellular enveloped virion are essential for virus spread.^6^, ^10^

The specific diagnostic test for monkeypox is polymerase chain reaction and it should be said that limited laboratories can perform this test. Virus propagation using cell culture allows further identification but is limited to reference laboratories with biosafety level 3.^11^ Serological testing can also be potentially useful in epidemiological research.^12^ Currently, there is no US Food and Drug Administration (FDA)‐approved treatment specifically for monkeypox. However, there are some general antiviral drugs, such as cidofovir, brincidofovir, and tecovirimat.^13^ In addition to antiviral agents, intravenous immunoglobulin has also previously been approved by the FDA for the treatment of complications from vaccinia vaccination.^14^ Unfortunately, no vaccine is designed for monkeypox, and the only vaccine intended for use is Vaccinia.^7^

Smallpox is estimated to have killed millions worldwide^15^ and was one of the most feared infectious diseases in human history. As a result of smallpox immunization programs that ended more than 40 years ago, a significant portion of the world's population is not immune to smallpox and zoonotic mpox.^16^ Due to the lack of anti‐monkeypox drugs and a specialized vaccine against this virus, the outbreak of monkeypox may be the beginning of another pandemic and may even be much more dangerous than Covid‐19. The aim of this research was to design synthetic antibodies against the monkeypox virus by in silico methods, such as docking and molecular dynamics simulation. In this study, a list of 100 designed antibodies was evaluated and among them, six of them with better characteristics were selected and introduced.

## 2 MATERIALS AND METHODS

### 2.1 Design of modeled antibodies against monkeypox virus

To design modeled antibodies, the sequence of a heavy chain of human antibody was first extracted from the NCBI (https://www.ncbi.nlm.nih.gov/protein/QGA67041.1), and then 10 amino acids were added to the beginning of this sequence. In this study, about 100 modeled antibodies with different sequences were designed and evaluated. It should be noted that the initial sequence had only 2 types of amino acids, including Lysine (K) and Glutamic acid (E). SWISS‐model server (https://swissmodel.expasy.org/) was used to prepare the three‐dimensional (3D) structure of the modeled antibodies. SWISS‐model, SAVES v6.0 (https://saves.mbi.ucla.edu/), and ProSA (https://prosa.services.came.sbg.ac.at/prosa.php) servers were also used to validate all antibody models.

### 2.2 Docking of synthetic antibodies and C19L protein

C19L is the most important surface protein in the monkeypox virus^17^ and was considered a target in this study. Here, as in the above step, the C19L sequence was first extracted from the NCBI (https://www.ncbi.nlm.nih.gov/protein/17529825), and then its 3D structure was prepared with SWISS‐model server and validated by SWISS‐model, SAVES v6.0, and ProSA servers. Then, docking was done with each modeled antibody and C19L protein by HDOCK online server (http://hdock.phys.hust.edu.cn/). Finally, six of them with the best docking energy and RMSD were selected.

### 2.3 Docking of modeled antibodies‐C19L complex and gamma Fc receptor type I

In this part of the work, the interaction of modeled antibodies‐C19L complex with gamma Fc receptor type I was evaluated. In this regard, 6 of the best‐modeled antibodies that had the maximum ability to bind to C19L protein were selected and docked separately with gamma Fc receptor type I (https://www.ncbi.nlm.nih.gov/protein/NP_001365733.1) by HDOCK online server.

### 2.4 Molecular dynamics simulation

In the next step, to check the stability of docking molecules during the simulation time, molecular dynamic simulation was applied by Ascalaph Designer software. Simulations were carried out in the NVT ensemble at 310 Kelvin for 5000 picoseconds. Finally, the changes in the free energy and RMSD were also monitored during the simulation.

### 2.5 Biological properties of modeled antibodies

Immunogenicity, allergenicity, and toxicity of modeled antibodies were obtained from Vaxijen (http://www.ddg-pharmfac.net/vaxijen/VaxiJen/VaxiJen.html), AllerTOP (https://www.ddg-pharmfac.net/AllerTOP), and ToxinPred (https://webs.iiitd.edu.in/raghava/toxinpred/design.php) servers, respectively. Also, Protparam server (https://web.expasy.org/protparam/) was used to calculate number of amino acids, molecular weight, theoretical pI, total number of negatively/positively charged residues, estimated half‐life, instability index, aliphatic index, and grand average of hydropathicity of modeled antibodies.

### 2.6 Experimental analysis by SPR and calculation of thermodynamic parameters

The interaction between C19L protein and anti‐C19L antibodies (wild‐type and synthetic) was evaluated by the SPR method. First, SPR surface was activated by *N*‐hydroxysuccinimide/*N*‐ethyl‐*N*′‐(3‐dimethylaminopropyl) carbodiimide hydrochloride and then immobilized by C19L. Here, the contact and dissociation time was 5 min and 20 min, respectively. Association rate (*k*_*on*_), dissociation rate (*k*_*off*_), and dissociation constant (*K*_D_) were calculated by a global fitting analysis assuming a Langmuir binding model.

(1) $$K_{D}=k_{off}/k_{on}$$

Also, changes in enthalpy (Δ*H°*) and entropy (Δ*S°*) were calculated from the slope and intercept, respectively.

(2) $$\;\ln\;K_{D}=\Delta H^{\circ }/RT+\Delta S^{\circ }/R$$

where *R* is the gas constant, and *T* is the absolute temperature.

The activation energy parameters were obtained from the temperature dependence of the association rate constant following the Eyring approximation.

(3) $$\ln(k_{on}/T)=-(\Delta H^{‡}/RT)+(\Delta S^{‡}/R)+\;\ln(k_{B}/h)$$

where *k*_*on*_ is the association rate constant, Δ*H* ^‡^ is the activation enthalpy, *R* is the gas constant, *T* is the absolute temperature, *ΔS* ^*‡*^ is the activation entropy, *k*_*B*_ is the Boltzmann's constant, and *h* is the Plank's constant.

## 3 RESULTS

### 3.1 Modeling and validation of designed antibodies, C19L protein, and gamma Fc receptor type I

**Figure 1:**
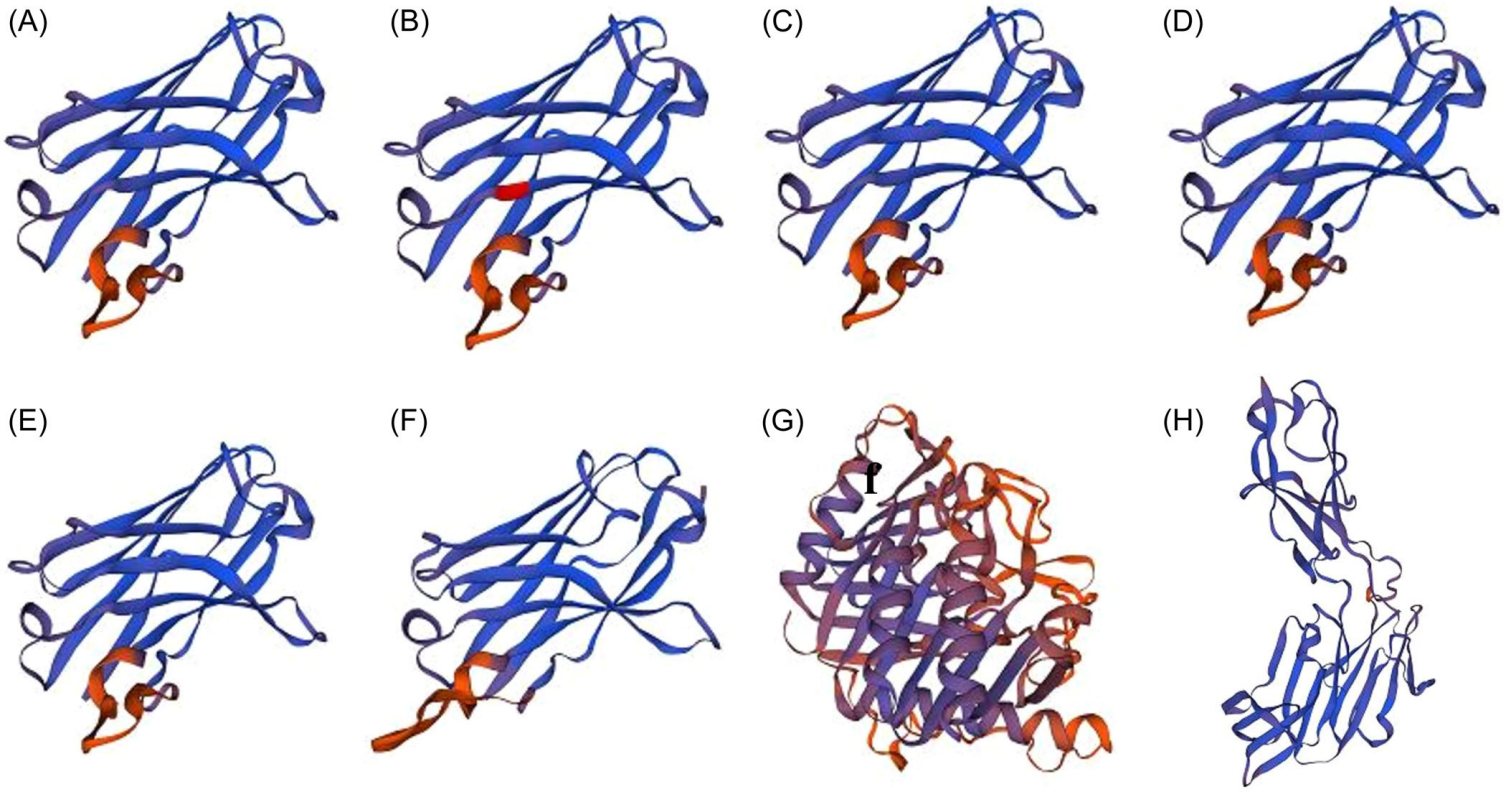
The three‐dimensional (3D) structure model of antibody 25 (A), antibody 28 (B), antibody 32 (C), antibody 37 (D), antibody 54 (E), antibody 62 (F), C19L (G), and gamma Fc receptor type I (H), prepared by SWISS‐model server.

Figure 1 shows the 3D structure model of antibodies (25, 28, 32, 37, 54, and 62), C19L, and gamma Fc receptor type I, prepared by SWISS‐model server. Overall model quality, local model quality, and surface energy of modeled antibodies are shown in Supporting Information S1–S3, respectively. Ramachandran Plots and QMEAN Z‐Scores of modeled antibodies are shown in Supporting Information S4 and S5, respectively. As can be seen, all 6 modeled antibodies were equally validated.

### 3.2 Docking and molecular dynamics simulation data

**Figure 2:**
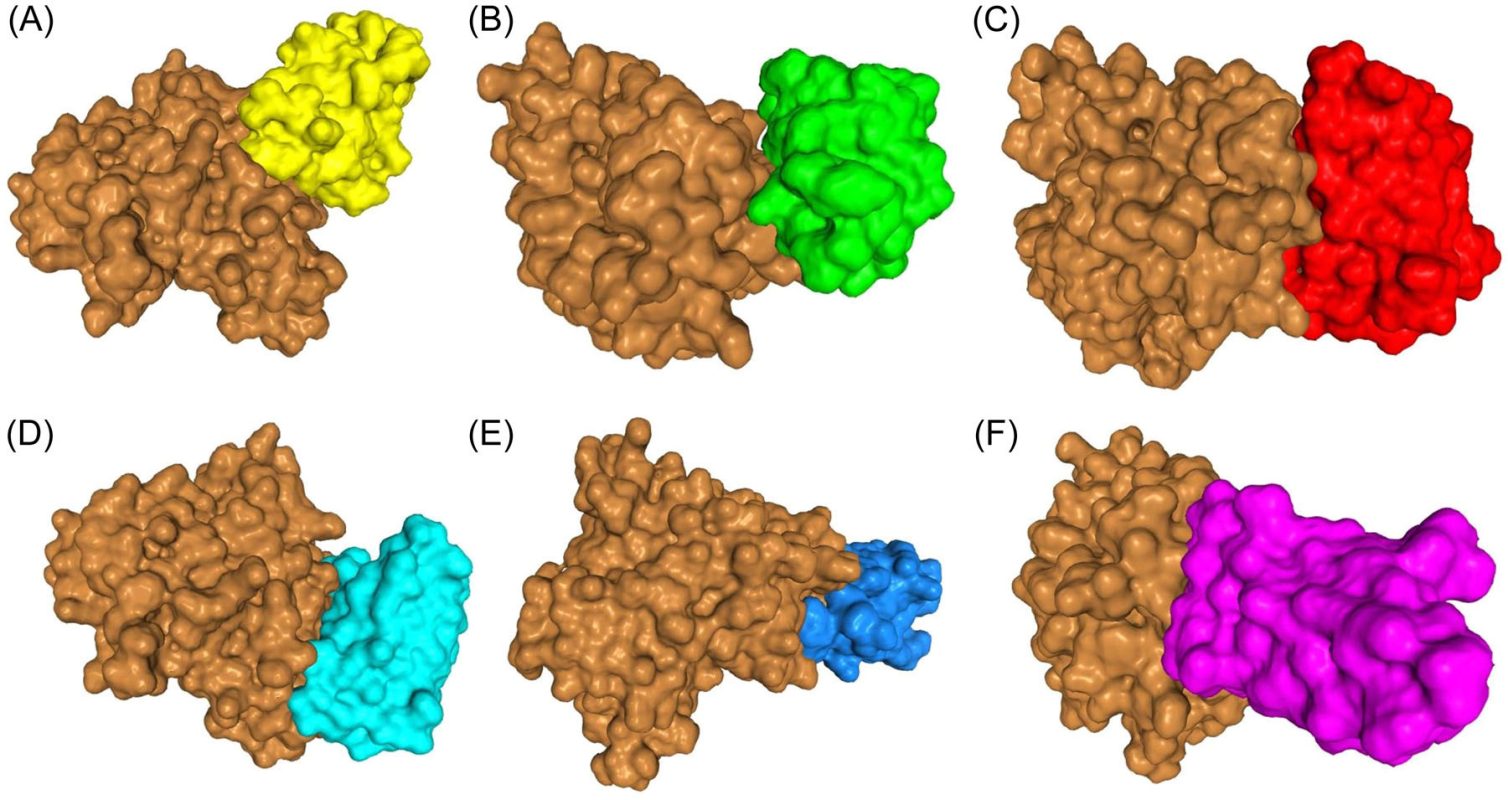
Docking of modeled antibody 25 (A), antibody 28 (B), antibody 32 (C), antibody 37 (D), antibody 54 (E), and antibody 62 (F) with C19L protein.

**Figure 3:**
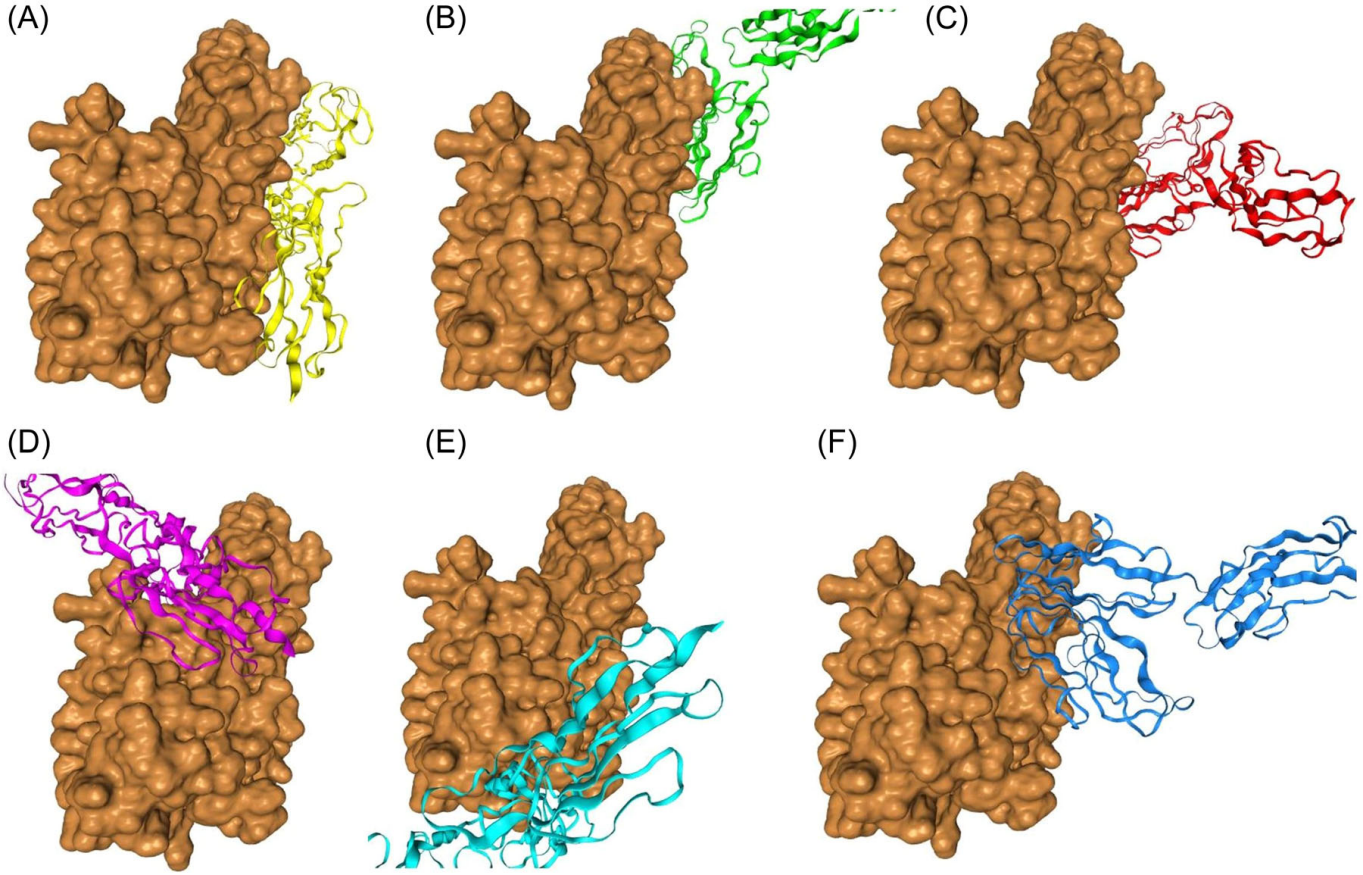
Docking of modeled antibody 25 (A), antibody 28 (B), antibody 32 (C), antibody 37 (D), antibody 54 (E), and antibody 62 (F)‐C19L complex with gamma Fc receptor type I.

**Figure 4:**
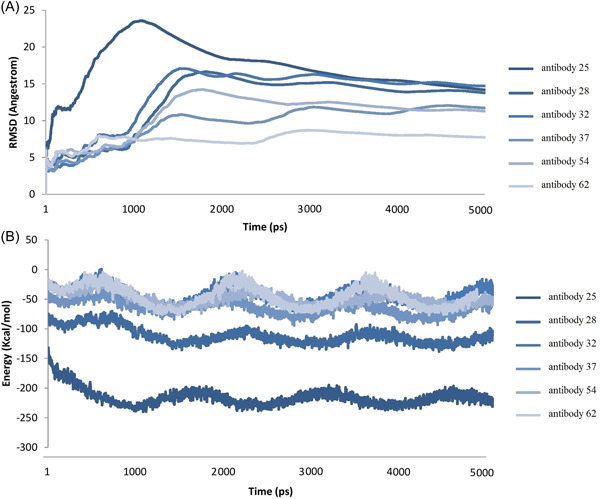
RMSD (A) and free energy (B) of modeled antibodies‐C19L complex during simulation time. RMSD, root‐mean‐square deviation.

**Figure 5:**
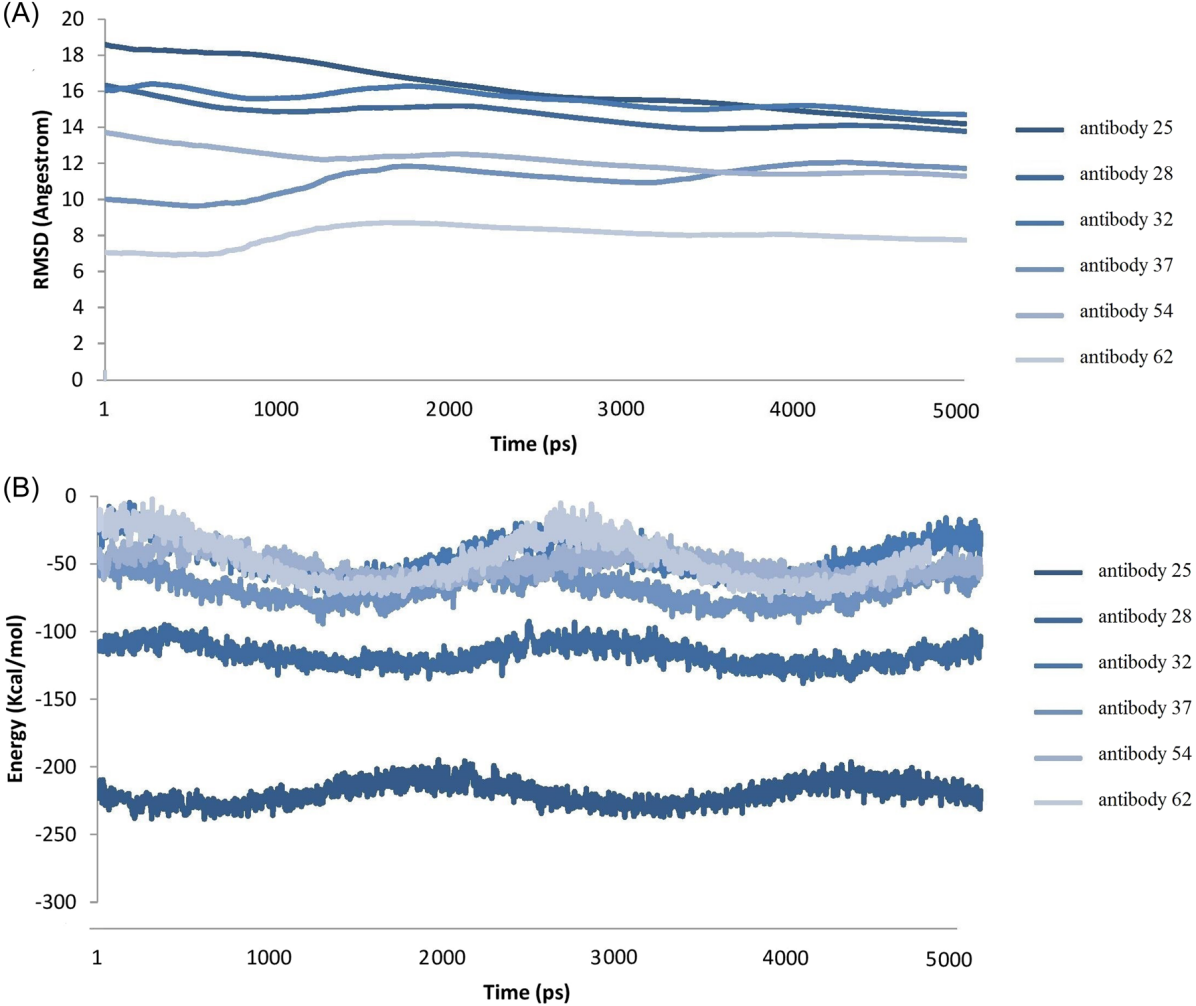
RMSD (A) and free energy (B) of modeled antibodies‐C19L‐gamma Fc receptor type I complex during simulation time. RMSD, root‐mean‐square deviation.

Figure 2 shows the docking of selected modeled antibodies with C19L protein. Their antibody number, sequence, docking energy, and RMSD are separately listed in Table 1. As can be seen, the range of docking energy and RMSD of selected modeled antibodies is from −124 to −154 kcal/mL and 4–6 angstrom, respectively. The best result was for antibody 62. Figure 3 shows docking of selected modeled antibodies‐C19L complex with gamma Fc receptor type I. Their docking energy and RMSD are separately listed in Table 1. Here, the range of docking energy and RMSD is from −132 to −155 kcal/mL and 5–7 angstrom, respectively. Here, the best result was also for antibody 62. Figure 4 shows RMSD (A) and free energy (B) of modeled antibodies‐C19L complex during simulation time. Also, Figure 5 shows RMSD (A) and free energy (A) of modeled antibodies‐C19L‐ gamma Fc receptor type I complex during simulation time. In both cases, the highest stability with the lowest energy level and RMSD was seen for antibody 62.

**Table 1:** The docking energy and RMSD of (modeled antibodies with C19L) and (modeled antibodies‐C19L complex with gamma Fc receptor type I).

		Docking of modeled antibodies with C19L		Docking of modeled antibodies‐C19L complex with gamma Fc receptor type I.	
Antibody number	Sequence	Docking energy (Kcal/mol)	RMSD (Å)	Docking energy (Kcal/mol)	RMSD (Å)
25	EKEKEEKKEKevqlvesggglvqpggslrlscaasgftfssyemnwvrqapgkglewvsyisssgstiyyadsvkgrftisrdnaknslylqmnslraedtavyycarewdggysgydsgdwyfdlwgrgtlvtvss#	−124	5	−132	5
28	EKKKEKKEKKevqlvesggglvqpggslrlscaasgftfssyemnwvrqapgkglewvsyisssgstiyyadsvkgrftisrdnaknslylqmnslraedtavyycarewdggysgydsgdwyfdlwgrgtlvtvss	−127	4	−135	5
32	KEEKEEKKEKevqlvesggglvqpggslrlscaasgftfssyemnwvrqapgkglewvsyisssgstiyyadsvkgrftisrdnaknslylqmnslraedtavyycarewdggysgydsgdwyfdlwgrgtlvtvss	−137	5	−135	6
37	KEKKKEKKEEevqlvesggglvqpggslrlscaasgftfssyemnwvrqapgkglewvsyisssgstiyyadsvkgrftisrdnaknslylqmnslraedtavyycarewdggysgydsgdwyfdlwgrgtlvtvss	−138	7	−147	7
54	EKEKKKKEEKevqlvesggglvqpggslrlscaasgftfssyemnwvrqapgkglewvsyisssgstiyyadsvkgrftisrdnaknslylqmnslraedtavyycarewdggysgydsgdwyfdlwgrgtlvtvss	−145	6	−152	7
62	KKEEEKEKKKevqlvesggglvqpggslrlscaasgftfssyemnwvrqapgkglewvsyisssgstiyyadsvkgrftisrdnaknslylqmnslraedtavyycarewdggysgydsgdwyfdlwgrgtlvtvss	−154	5	−155	5

Abbreviation: RMSD, root‐mean‐square deviation.

# The sequence of a heavy chain of human antibody was first extracted from the NCBI, and then 10 amino acids (yellow highlighted) were added to the beginning of this sequence.

### 3.3 Biological properties of modeled antibodies

Table 2 shows the biological properties of modeled antibodies. Interestingly, no one had immunogenicity, allergenicity, and toxicity. Although all the modeled antibodies had good stability, only antibodies 25, 28, 54, and 62 had a half‐life of >10 h. Other biological parameters were almost the same for all.

**Table 2:** Biological properties of modeled antibodies.

	Antibody number					
	25	28	32	37	54	62
Immunogenicity	Not antigenic	Not antigenic	Not antigenic	Not antigenic	Not antigenic	Not antigenic
Allergenicity	Not allergic	Not allergic	Not allergic	Not allergic	Not allergic	Not allergic
Toxicity	Not toxin	Not toxin	Not toxin	Not toxin	Not toxin	Not toxin
Number of amino acids	137	137	137	137	137	137
Molecular weight	15,251.82	15,249.94	15,251.82	15,250.88	15,250.88	15,250.88
Theoretical pI	5	7.97	5.03	5.53	5.53	5.53
Total number of negatively charged residues	18	16	18	17	17	17
Total number of positively charged residues	15	17	15	16	16	16
Estimated half‐life	>10 h	>10 h	3 min	3 min	>10 h	>10 h
Instability index	36.21	33.83	38.59	38.59	36.21	38.59
Aliphatic index	61.90	61.90	61.90	61.90	61.90	61.90
Grand average of hydropathicity	−0.558	−0.564	−0.558	−0.561	−0.561	−0.561

### 3.4 The kinetic and thermodynamic parameters

Table 3 shows the kinetic and thermodynamic parameters of binding due to interaction between C19L protein and anti‐C19L antibodies (wild‐type and synthetic). As can be seen, K_D_ in synthetic antibodies was lower than wild antibody and the lowest value was seen for antibody 62. In terms of δH°, TδS°, and δG°, the results were consistent with binding parameters. Here, the lowest value of thermodynamic parameters was obtained for antibody 62. These data show that the synthetic antibodies, especially antibody 62, had a higher affinity than the wild‐type antibody.

**Table 3:** The kinetic and thermodynamic parameters of binding due to interaction between C19L protein and anti‐C19L antibodies (wild‐type and synthetic).

Antibody number	K_on_ (S^‐1^)	K_off_ (S^‐1^)	K_D_(nM)	K_D_(WT)/K_D_(synthetic)	δH^o^ (kcal/mol)	‐TδS^o^ (kcal/mol)	δG^o^ (kcal/mol)
WT	130,000	104,000	0.8	1	−7	−5	−12
25	130,000	91,000	0.7	1.4	−9	−5	−12.3
28	140,000	84,000	0.6	1.3	−10	−8	−12.4
32	70,000	21,000	0.3	2.6	−12	−9	−12.4
37	150,000	75,000	0.5	1.6	−18	−9	−12.4
54	130,000	26,000	0.2	4	−24	−10	−13
62	100,000	10,000	0.1	8	−25	−12	−14

## 4 DISCUSSION

Today, gamma immunoglobulins (IgG) are used in the treatment of many diseases. IgG molecules can bind to specific antigens, and then the antibody‐antigen complex can interact with Fc receptors, located on innate and acquired immune cells.^18^ Structurally, IgG consists of four polypeptide chains, including two light chains (LC) and two heavy chains (HC). These four chains create two Fab domains to bind to the antigen and one Fc domain to bind to Fc receptors.^19^ Fab domains have variable and constant domains. Variable domains, especially with CDRs, are mainly responsible for specificity and affinity,^20^ while constant domains give rise to isotypes.^21^ The Fc domain itself contains two CH2 and CH3 sub‐domains. The CH2 sub‐domain mainly interacts with Fc receptors that are on the cell surface. Other regions, including hinge and glycan regions, also affect antibody activity.^22^ The constant domain of antibodies also plays a role in the detection of antigens.^23^ Antibodies with identical constant domains exhibit differences in affinity^24^ or specificity.^25^ The good news is that all parts of antibodies can be engineered using different methods to obtain better properties. Fortunately, with computational methods, it is possible to model antibodies and predict their behavior. Algorithms and force fields are becoming more accurate and can be used to engineer antibodies.^26^ CDRs are part of variable domains that play a central role in the design and engineering of antibodies.^27^ It is interesting to say that CDRs are in human and mouse antibodies, but not in other organisms.^28^

The ab initio methods can be used for antibody modeling.^29^ PLOP,^30^ Modeller,^31^ Loopy,^32^ and Rosetta^33^ are some examples of modeling software or servers. Antibody modeling is done using physicochemical properties instead of using structural models. To evaluate the reliability of predictions, modeling data must be compared with experimental data. Although much progress has been made to predict antibody structure, experimental methods still have higher quality and accuracy. By using 3D structures of antibody‐antigen complexes and changing their sequence, the affinity of an antibody can be changed. If the 3D structure of the antibody‐antigen complex is available, it is relatively simple to engineer antibodies to increase the affinity. The sequence of antibodies can be changed. For example, Kiyoshi et al.^34^ investigated antibody mutations and confirmed them by in silico data. Among 12 mutants, five of them had increased binding affinity. In another study, a 10‐fold increase in antibody affinity was reported by in silico antibody design.^35^ Their results showed that the calculation of electrostatic energy can be a more accurate indicator to predict binding affinity between antibody and antigen.

As a result of smallpox immunization programs that ended more than 40 years ago, a significant portion of the world's population is not immune to smallpox and the zoonotic mpox.^16^ Due to the lack of anti‐monkeypox drugs and a specialized vaccine against this virus, the outbreak of monkeypox may be the beginning of another pandemic. Here, our purpose was to design a novel antibody against the monkeypox virus based on a heavy chain of human antibody and a small peptide sequence at the beginning of it. In this study, a list of 100 modeled antibodies was evaluated, and among them, six of them with better characteristics were selected and introduced. Docking of modeled antibodies with C19L protein showed the range of docking energy, and RMSD was from −124 to −154 kcal/ml and 4–6 angstrom, respectively. Also, docking of modeled antibodies‐C19L complex with gamma Fc receptor type I confirmed the results. Molecular dynamics simulation also showed that antibody 62 had the highest stability with the lowest energy level and RMSD. We found that K_D_ in synthetic antibodies was lower than wild antibody. In terms of δH°, TδS°, and δG°, the results were consistent with binding parameters. These data show that the synthetic antibodies, especially antibody 62, had a higher affinity than the wild‐type antibody.

One of the important challenges in the design of antibodies is the docking of antibodies and antigens. Since epitopes and paratopes are usually planar, conventional docking methods may not be suitable. SnugDock or RosettaDock are better choices because they rely on ensemble stochastic perturbation and generate a large number (~105) of models to capture the minimum energy.^36^ For example, Zhao et al used RosettaDock to design antibodies against Aβ/fibrils.^37^ Another method to model antibodies and antigens is the knowledge‐based residue pair preference on epitope‐paratope interfaces. Wang et al studied the physicochemical properties of antibodies and antigens, such as net charge, overall charge distribution, and their role in antigen interactions. They found that the selection of amino acids is based on entropy of antigen recognition. Amino acids with a positive and polar charge have a greater effect on antibody‐antigen recognition.^38^ Tharakaraman and colleagues succeeded to increase antibody affinity up to 450 times. This antibody could neutralize the dengue virus.^39^ Although there are many successful cases to increase the affinity of antibodies, there are still challenges. The effect of solvent and trapped water molecules is one of these challenges. At high concentrations, antibodies can accumulate,^40^ and their accumulation can lead to immunogenicity.^41^ Several experimental studies have been conducted to investigate the stability of antibodies.^42^ Interestingly, molecular modeling can be a useful tool to predict the accumulation of antibodies. Hydrophobicity, net charge, and secondary structure propensity can be useful to predict antibody aggregation.^43^ In several studies, MD simulation has been used to study antibody aggregation.^44^

As a last word, we must say that this dangerous virus will come to human society, and ways to deal with it should be prepared from now. The mechanisms related to this virus should be known and drugs or vaccines should be designed for it.^45^, ^46^, ^47^, ^48^, ^49^, ^50^ This study opened a small window to deal with this virus, and we hope that this way will continue.

## 5 CONCLUSION

Due to the lack of anti‐monkeypox drugs and a specialized vaccine against this virus, the outbreak of monkeypox may be the beginning of another pandemic. In this study, novel antibodies against monkeypox virus were modeled based on a heavy chain of human antibody and a small peptide sequence at its beginning. Docking of modeled antibodies with C19L protein showed the range of docking energy, and RMSD was from −124 to −154 kcal/mL and 4–6 angstrom, respectively. Also, docking of modeled antibodies‐C19L complex with gamma Fc receptor type I confirmed the results and showed the best result was for antibody 62. Molecular dynamics simulation also showed that antibody 62 had the highest stability with the lowest energy level and RMSD. No modeled antibodies had immunogenicity, allergenicity, and toxicity. Although all the modeled antibodies had good stability, only antibodies 25, 28, 54, and 62 had a half‐life of >10 h. It was found that K_D_ in synthetic antibodies was lower than wild antibody. In terms of δH°, TδS°, and δG°, the results were consistent with binding parameters. These data show that the synthetic antibodies, especially antibody 62, had a higher affinity than the wild‐type antibody.

## AUTHOR CONTRIBUTIONS

**Sadeq Shabani**: Visualization; writing—original draft; writing—review & editing. **Mohsen Rashidi**: Conceptualization; validation. **Shakila Radgoudarzi**: Supervision; validation; visualization.

## CONFLICTS OF INTEREST STATEMENT

The authors declare no conflicts of interest.

## ETHICS STATEMENT

Not applicable. This study was an in silico study.

## Supporting information

Supporting information.

Supporting information.

## Data Availability

All data of this article is available based on the official request of researchers.
