# Supplementary figures and images for: The validation of artificial anti‐monkeypox antibodies by in silico and experimental approaches
Source: Immun Inflamm Dis. 2023 Apr 12;11(4):e834. doi: 10.1002/iid3.834 (PMC10091375; doi:10.1002/iid3.834)

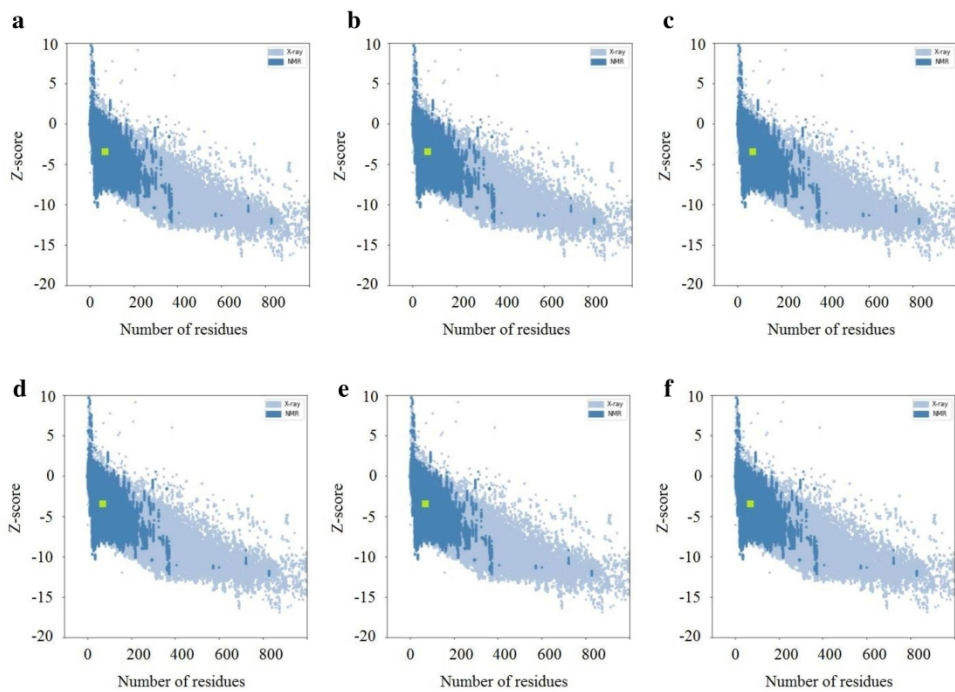

254x190mm (300 x 300 DPI)

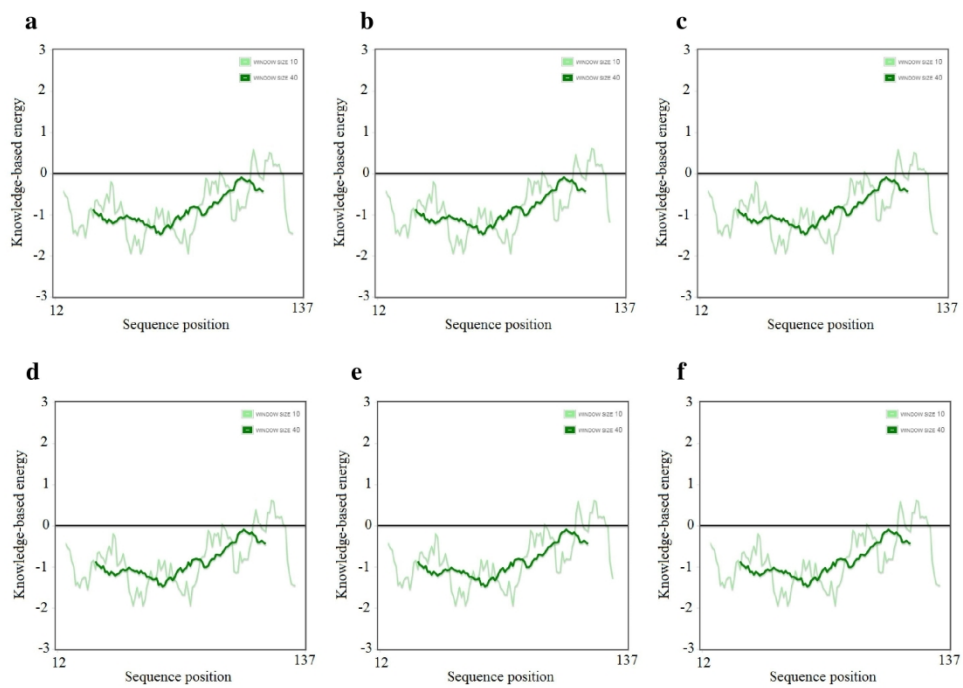

254x190mm (300 x 300 DPI)

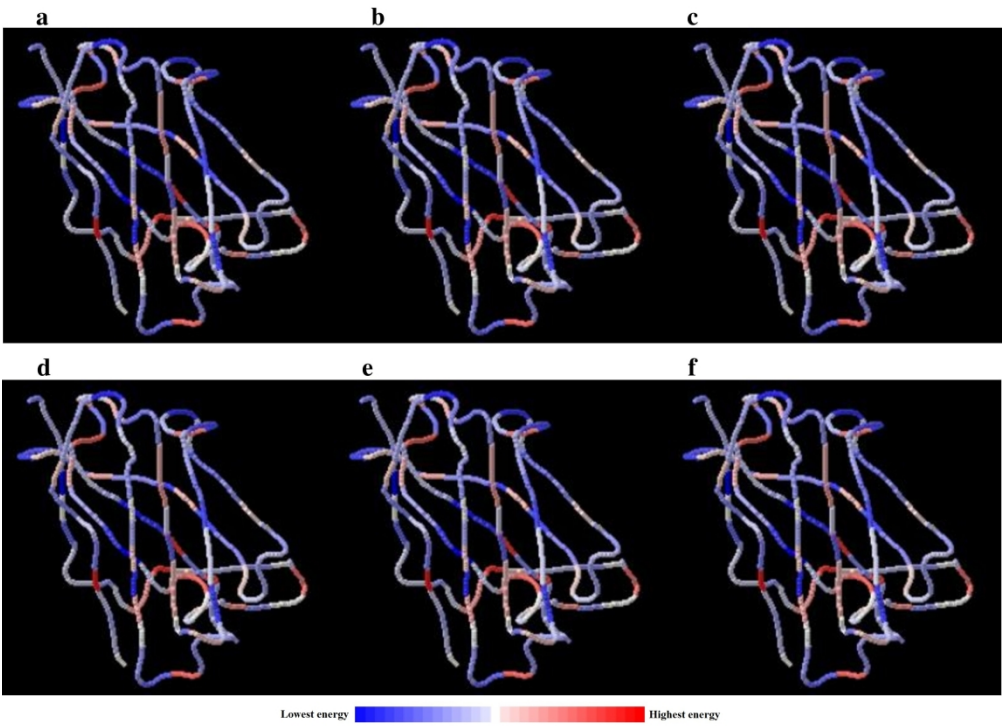

254x190mm (300 x 300 DPI)

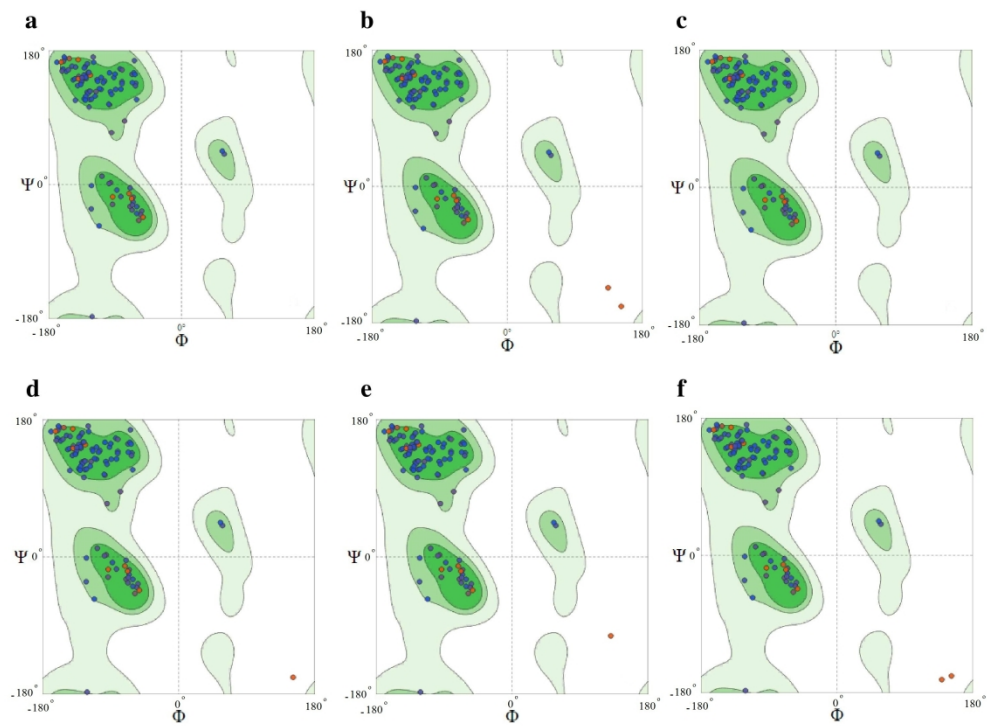

254x190mm (300 x 300 DPI)

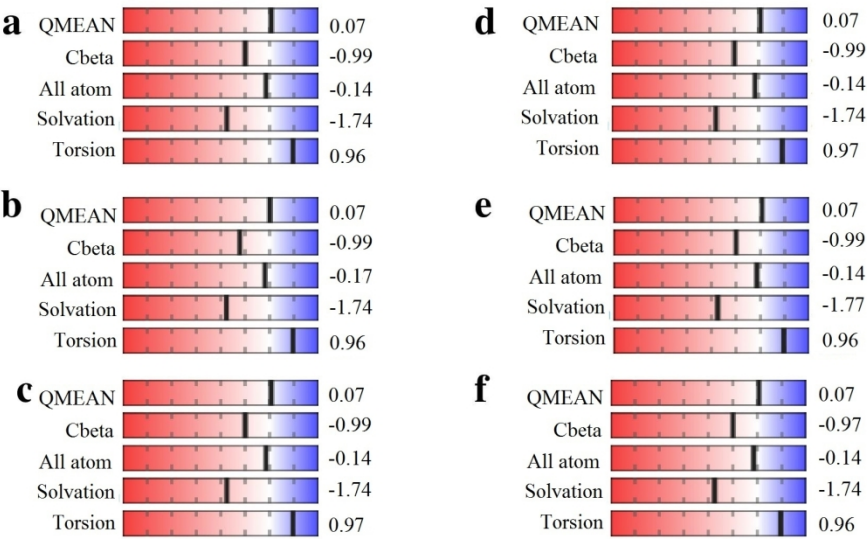

162x94mm (300 x 300 DPI)

Supplement: Supplementary file 1 — Supporting information. [file IID3-11-e834-s002.pdf]
