# Supplementary material for: The validation of artificial anti‐monkeypox antibodies by in silico and experimental approaches
Source: Immun Inflamm Dis. 2023 Apr 12;11(4):e834. doi: 10.1002/iid3.834 (PMC10091375; doi:10.1002/iid3.834)
Supplement: Supplementary file 2 — Supporting information. [file IID3-11-e834-s001.docx]

**Supplementary** **1.** Overall model quality of modeled antibody 25 (**a**), antibody 28 (**b**), antibody 32 (**c**), antibody 37 (**d**), antibody 54 (**e**), antibody 62 (**f**).

**Supplementary** **2.** Local model quality of modeled antibody 25 (**a**), antibody 28 (**b**), antibody 32 (**c**), antibody 37 (**d**), antibody 54 (**e**), antibody 62 (**f**).

**Supplementary** **3.** Surface energy of modeled antibody 25 (**a**), antibody 28 (**b**), antibody 32 (**c**), antibody 37 (**d**), antibody 54 (**e**), antibody 62 (**f**).

**Supplementary** **4.** Ramachandran Plots of modeled antibody 25 (**a**), antibody 28 (**b**), antibody 32 (**c**), antibody 37 (**d**), antibody 54 (**e**), antibody 62 (**f**).

**Supplementary 5.** QMEAN Z-Scores of modeled antibody 25 (**a**), antibody 28 (**b**), antibody 32 (**c**), antibody 37 (**d**), antibody 54 (**e**), antibody 62 (**f**).
